# A Disease Pathway Framework for Pain Point Identification and Elaboration of Product Requirements Across Patient Care Plan Using Innovation Think Tank Global Infrastructure

**DOI:** 10.3389/fpubh.2022.862384

**Published:** 2022-04-13

**Authors:** Sultan Haider, Apoorva Goenka, Mohd Mahmeen, Shamlin Sunny, Thuong Phan, Syed Ali Mehdi, Dahlia Mohamed Hassan, Elena Weber

**Affiliations:** ^1^Innovation Think Tank, Siemens Healthcare GmbH, Erlangen, Germany; ^2^Innovation Think Tank, Siemens Healthcare Pvt. Ltd., Bengaluru, India; ^3^Innovation Think Tank, Siemens Healthcare GmbH, Kemnath, Germany; ^4^Innovation Think Tank, Siemens Medical Solutions Inc., Princeton, NJ, United States; ^5^Innovation Think Tank, Siemens Healthcare LLC, Dubai, United Arab Emirates

**Keywords:** disease pathways, Innovation Think Tank, care plans, chronic disease management, disease statistics, healthcare system

## Abstract

Healthcare providers as well as medical technologists lay a strong focus on clinical conditions for patient centric care delivery. Currently, the challenges are to (1) obtain a consolidated view of various stakeholders and pain points for the entire disease lifecycle, (2) identify interdependencies between different stages of the disease, and (3) prioritize solutions based on customer needs. A structured approach is required to address clinical needs across disease care plans tailored to different geographies and ethnicities. Innovation Think Tank (ITT) teams across multiple locations formed focus groups to elaborate the pathways of 22 global diseases, selected based on ranking of associated economic burden and threat to life. Ideation sessions were held to identify pain points and find innovative solutions. Additionally, inputs were taken from co-creation sessions at universities worldwide. The optimization and design of infographics and care plan was done based on the key information gathered—facts and figures, stakeholders, pain points and solutions. Finally, validation was obtained from clinical and technology experts globally. A disease pathway framework was created to develop pathways for 22 global diseases. Over 1,500 pain points were collected and about 1,900 ideas were proposed. The approach was applied to optimize its application to 30 product and portfolio definition projects over 2 years at Siemens Healthineers, as well as co-creation programs with universities and hospitals. The disease pathway framework provides a unique foundation for extensive collaboration among multiple stakeholders, through information sharing and delivering high-quality solutions based on the identified problems and customer needs.

## Introduction

**Figure 1:**
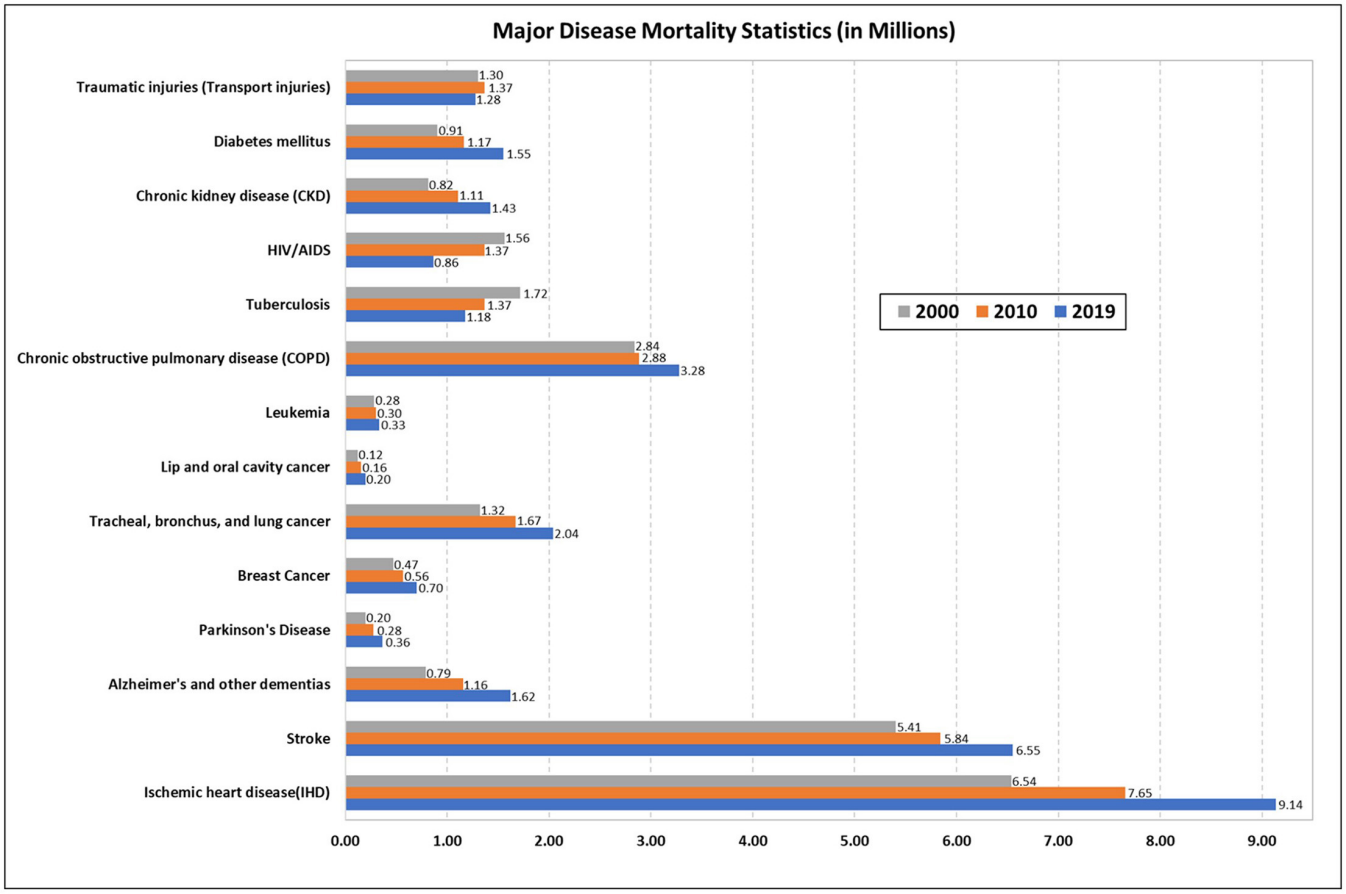
Major disease mortality statistics from 2000, 2010, 2019.

The Global Burden of Disease study addresses the changing healthcare needs of the global population from 1990 to 2020. It is estimated that as the population ages, the disease burden will shift from communicable diseases to non-communicable diseases (NCDs) and injuries, and most deaths and disabilities will be due to the latter (1). Fifty percent of the world's population does not have access to basic health services. Due to rising healthcare expenditure, millions of people are pushed into extreme poverty (2). Chronic diseases may result in increased treatment costs due to presence of comorbidities, readmissions, diagnostic and medication expenses. It may also cause decreased productivity due to disability. Moreover, increasing disease burden can lead to delayed diagnosis, inappropriate treatment, and increase in mortality and morbidity. Disease burden could be categorized by various health-related indicators like mortality indicators, morbidity indicators, disability rates, and socio-economic indicators (3, 4). It is critical to understand why individuals die since mortality data (Figure 1) can help us evaluate the efficiency of our health systems and allocate appropriate resources to different sectors, responding to varying epidemiological situations effectively (5, 6).

According to a World Health Organization (WHO) report, of the 55.4 million deaths worldwide in 2019, 55% were due to the ten leading causes of death. NCDs were responsible for 74% of deaths worldwide in 2019. The worldwide economic burden of NCDs is projected to be around $47 trillion from 2010 to 2030 (7). Cardiovascular diseases (CVDs) are the leading cause of premature deaths and growing healthcare expenditure which accounts for nearly 50% of the economic disease burden (8). Due to exponential population growth, aging population and changing prevalence of risk factors, the burden of cancer is increasing worldwide (19.3 million new cases and 10 million deaths in 2020) (9). The economic loss from cancer related disability and death worldwide was $895 billion in 2008 (10). In the US, $246 billion is projected to be spent on cancer care in 2030 (11). Morbidity, mortality and disability due to neurological diseases, chronic respiratory diseases, chronic kidney disease (CKD) and diabetes are also on the rise (12–16). In the US alone, neurological disorders contributed to an economic burden of $800 billion in 2014 (17). In 2019, healthcare cost due to chronic respiratory diseases (CRDs) in the EU region alone was 380 billion Euros (14, 15). 6.7 million died and US$ 966 billion was the healthcare spending due to diabetes or its complications in 2021. It is estimated to reach US$ 1 trillion by 2030 (16). Ischemic heart disease (IHD), stroke, and chronic obstructive pulmonary disease (COPD) accounted for 16, 11, and 6% of global deaths, respectively, in 2019 (18). There has been an overall decrease in the burden of communicable diseases (CDs), evident from the fall in the incidence and deaths due to tuberculosis and HIV over the years (19, 20). Apart from NCDs and CDs, injuries are also responsible for many deaths globally. According to the WHO “Global status report on road safety 2018,” road traffic crashes result in 1.35 million annual deaths (21).

Global chronic disease burden is remarkable, and the conventional methods for healthcare delivery are unsuitable to address the current needs. In the complex chronic disease healthcare environment, we have different stakeholders and their interdependencies throughout different stages of the disease (22). Major value providers responsible for care delivery are medical device manufacturers and clinicians. These two stakeholders are interdependent, working to understand the problem, design solutions, and deliver appropriate care to the patient. So, on both sides there is a need to understand the chronic disease management stages to reach, assess and solve the challenges of the patients. Most education about health care during the progression of chronic disease is through the introduction of preventive measures, which are strongly associated with risk factors (23). Though not an illness, risk factors need to be identified and managed in asymptomatic people as it might lead to complications in some individuals later in life (24). It is imperative to focus on health promotion, raise awareness, modify health behaviors, and promote early disease screening to facilitate disease prevention (25, 26). Physical symptoms account for fifty percent of all out-patient visits. Educating individuals about symptoms of a particular disease can contribute to prompt assessment, early intervention, and improved patient outcome (27). Moreover, disease management can be improved by early diagnosis and timely treatment, thereby preventing complications and prolonging patient survival. Patients with chronic disease often require rehabilitation to get back to their daily routine and have a healthy lifestyle. Emphasis is now placed on evidence-based care by rehabilitation clinicians as it is an essential part of patient care (28–30).

From the above literature review, we can conclude that to achieve optimal patient care, we need a structured approach to address clinical needs across disease care plans globally. Looking at the bigger picture, what is required is sustainable innovation ecosystem and technological advancement using structured, dynamic and customer centric frameworks to drive progress in the healthcare system globally. It is challenging to get a consolidated perspective of diverse stakeholders and pain points for the entire disease lifecycle, and to identify interdependencies between different stages of disease. It is also difficult to prioritize solutions as per customer needs. We thus proposed a framework for disease pathways to provide an overall picture of where the difficult points lie in the disease management and to propose solutions for the same. The disease pathway is a novel tool that describes the procedures involved in healthcare delivery throughout the development of various diseases. The framework was applied for 22 diseases: coronary artery disease (CAD), stroke, structural heart disease, peripheral vascular disease (PVD), AD, dementia, PD, multiple sclerosis (MS), depression, lung cancer, breast cancer, liver cancer, oral cancer, leukemia, COPD, CKD, diabetes, TB, HIV, infectious diseases, traumatic injury, and wound healing disorders. With the focus on open innovation in healthcare, the complete disease pathways, comprising pain points, impact of COVID-19 and possible solutions on emerging technologies to address the real-world challenges, have been curated and validated with clinical experts globally.

## Methods

With over 16 years of experience, Innovation Think Tank (ITT) teams have developed real-world healthcare models that incorporate observed “best practices” gathered from 900+ global hospital visits, multiple co-creation sessions with clients, and literature reviews. Each hospital department is visualized, and information maps of observed problems, also called “pain points,” and best practices are placed at the appropriate location in the model (31, 32). This data provides a valuable basis for visualizing areas where the acquired mission can be identified and workflows that can benefit from an innovative solution or product. ITT introduces different stakeholders and brings them together under the same mandate to create something together. However, it is crucial to note that healthcare facilities differ from the regions in which they are located. Therefore, it is essential to conduct evaluations in regional contexts.

We identified 22 global diseases based on the highest economic burden, social impact, and mortality rate. To create the disease pathway framework, there was a need of a suitable methodology which could cover all qualitative and quantitative aspects. “Sultan Haider in 2005” developed an ITT methodology which contains steps such as acquiring a mandate, identifying the problem, analyzing the root cause, identifying interdependencies, engaging stakeholders, creating the big picture, conceptualizing, and validating with experts, creating decision propositions, and commercializing the solution (31). This methodology was suitable because it addresses the problem, includes all stakeholders. Above steps in ITT methodology follows a mixed approach with qualitative and quantitative aspects. As mentioned, taking the above methodology as a reference, ITT teams around the world formed groups. Disease topics were assigned to each group based on members' skills and areas of interest. 20 co-creation sessions were held within a transdisciplinary team of more than 50 participants composed of medical experts from ITT sites worldwide and ITT's technical and biomedical team to understand the disease, develop the disease care plan, and analyze pain points during the disease life cycle.

**Figure 2:**
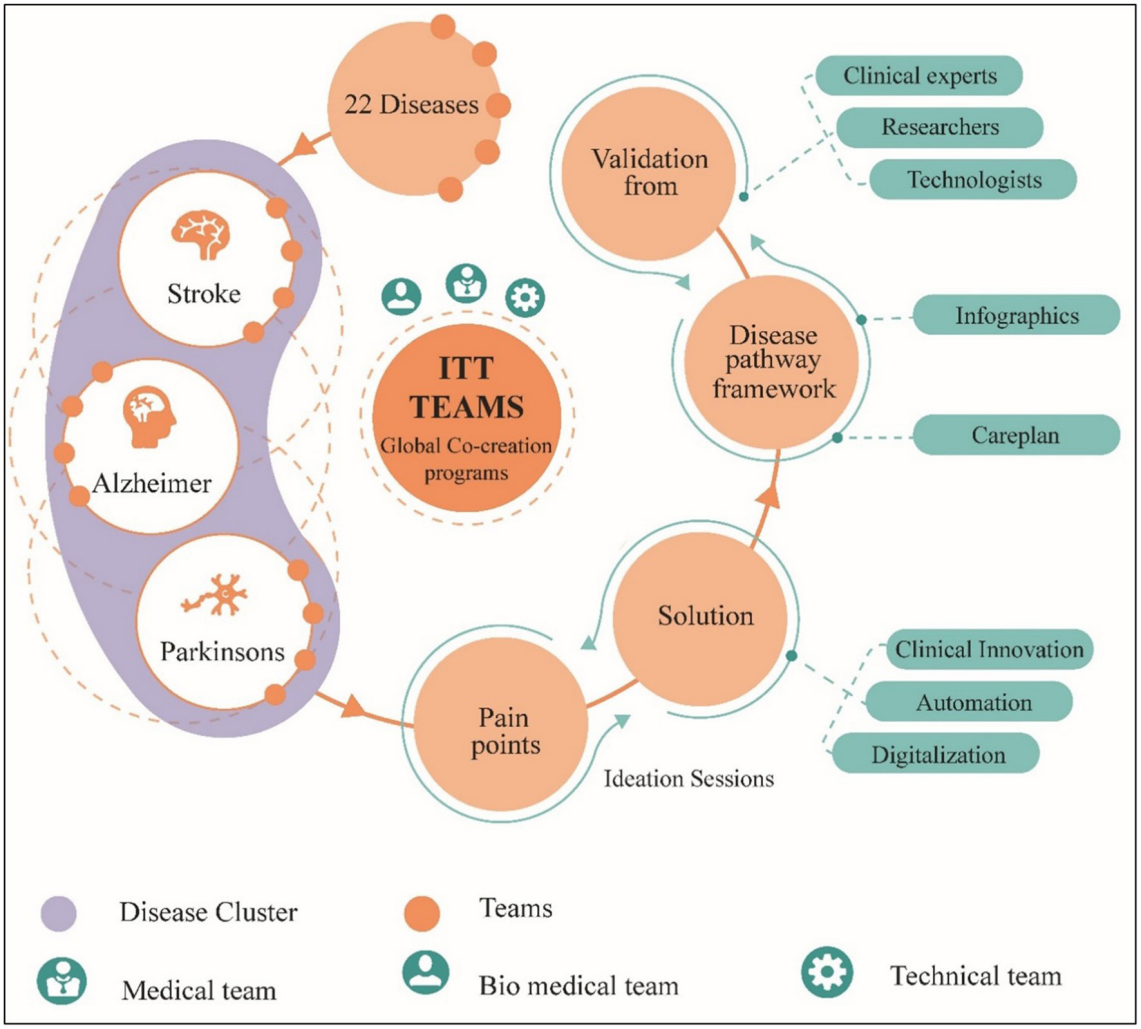
Transdisciplinary methodology to establish disease pathway framework by identifying pain points, proposing solutions, and validating by clinicians.

Disease clusters were formed, and topics were rotated between groups to gather insights across all disciplines. Ideation sessions were organized for each pain point to identify innovative solutions in the categories of digitization, automation, and clinical innovation while team members swapped roles. Additionally, inputs were taken from co-creation programs at universities in Germany, the United States of America (USA), United Kingdom (UK), India, China, Turkey, United Arab Emirates (UAE), and South Africa. The optimization and design of the infographic and care plan was done for each disease based on the key information gathered—facts and figures, stakeholders, pain points and associated solutions. Finally, extensive validation of the same was obtained from experts worldwide (Figure 2). The ITT co-development and laboratory infrastructure program supported the adaptation of these disease pathways, considering global trends and challenges. In 2020, after the pandemic COVID-19, the disease pathways were adapted to the impact of SARS-CoV-2.

Two significant features of these disease pathways are to showcase impact on global & economic burden and inclusion of stages before the onset of each disease. Health care providers believe that prevention is always better than cure, and therefore disease trajectories are presented in eight stages: prenatal, prevention, symptoms, diagnosis, treatment, rehabilitation, follow-up and out-patient. Looking at the disease trajectories in their entirety enables the creation of requirements for solutions that address the challenges holistically and help stakeholders prevent or halt the progression of the disease.

## Results

A disease pathway framework was created to develop disease pathways for 22 global diseases. The proposed framework consists of three core elements as follows: (1) Facts and figures template (2) Disease pathway (3) Technological solution framework.

In this article, we have covered the facts and figures template and the disease pathway. The technological solutions framework is out of the scope of this article. It will be considered in future work to present the disease-centric automation framework. Let us start with the facts and figures template. It is intended to provide a general and consolidated overview of common disease symptoms, followed by statistical figures on major diseases to show current incidence and mortality with their global and economic impact. Given the wealth of experience and impact of COVID-19 on chronic disease management, we have also made provisions to present the impact of community diseases.

We divided these selected diseases into clusters based on the International Statistical Classification of Diseases and Related Health Problems - 10 (ICD-10), a universally accepted medical classification system (Table 1).

**Table 1:** Disease clusters for the 22 global diseases considered in the study, based on the ICD-10 classification system.

**Disease cluster**	**Diseases**
Certain infectious and parasitic diseases	Infectious diseases Tuberculosis Human immunodeficiency virus/acquired immunodeficiency syndrome (HIV/AIDS)
Neoplasms	Lung cancer Breast cancer Liver cancer Oral cancer Leukemia
Endocrine, nutritional, and metabolic diseases	Diabetes
Mental and behavioral disorders	Depression
Diseases of the nervous system	Alzheimer's disease Dementia Parkinson's disease Multiple sclerosis
Diseases of the circulatory system	Stroke Coronary artery disease Structural heart disease Peripheral vascular disease
Diseases of the respiratory system	Chronic obstructive pulmonary disease
Diseases of the genitourinary system	Chronic kidney disease
Injury, poisoning and certain other consequences of external causes	Traumatic injury Wound healing disorder

Thereafter, a care plan was framed for every disease at each stage of development. Each care plan stage contains information about the stakeholders involved, the pain points, and the respective solutions. The disease care plan model presented is broadly divided into stages such as:

1. Prenatal: This stage is unique as it covers that portion of the disease life cycle that can occur or exist even before birth. It is important because by focusing on this stage, we can investigate techniques by which pregnant women can reduce the disease risk for their unborn child.
2. Prevention: How an ordinary person can prevent the occurrence of a particular disease is the focus of this section. Measures that can be taken to prevent the disease based on the risk factors have been elaborated in this stage for each disease. It also includes steps to stop the progression of a disease at an early stage to prevent it from getting worse.
3. Symptoms: This section mainly covers the disease's life cycle when the patient begins to feel the physical or psychological effects of the disease. Symptoms may or may not be disease specific. They play an essential role in the progression of the disease, which should never be neglected. The study of symptoms not only provides information on the onset of the disease but also aids in deciding on the course of treatment.
4. Diagnosis: The section of diagnosis mainly deals with identification of the nature of illness or other problems by clinical, laboratory and radiological diagnosis. Early diagnosis of chronic diseases is crucial because early detection leads to better treatment outcomes than intervening at a later stage. Therefore, different types of diagnostic tools and methods have been developed to correctly diagnose and determine the severity and subtype of the disease.
5. Treatment: The treatment section includes the medical or surgical care given to a patient for an illness or injury. As with diagnosis, a variety of existing and advanced therapeutic measures have been found to treat disease.
6. Rehabilitation: Rehabilitation mainly includes the interventions done to bring one back to the daily routine after an illness. It can include routine health monitoring and exercises in gym or sports centers, rehabilitation centers, using fitness equipment, devices, and following health plans.
7. Follow-up: This section comprises of care that the patient receives over a period after treatment completion. Regular medical checkups, clinical examination, laboratory and radiological tests, medication change might be included here.
8. Out-patient: This section includes the patient consultation that does not require overnight stay at the hospital. Patient history taking, physical examination, diagnostic tests, and treatment (medication, day care surgeries) can be included. Patient self-care and management at home is also considered in this section of the care plan.

**Figure 3:**
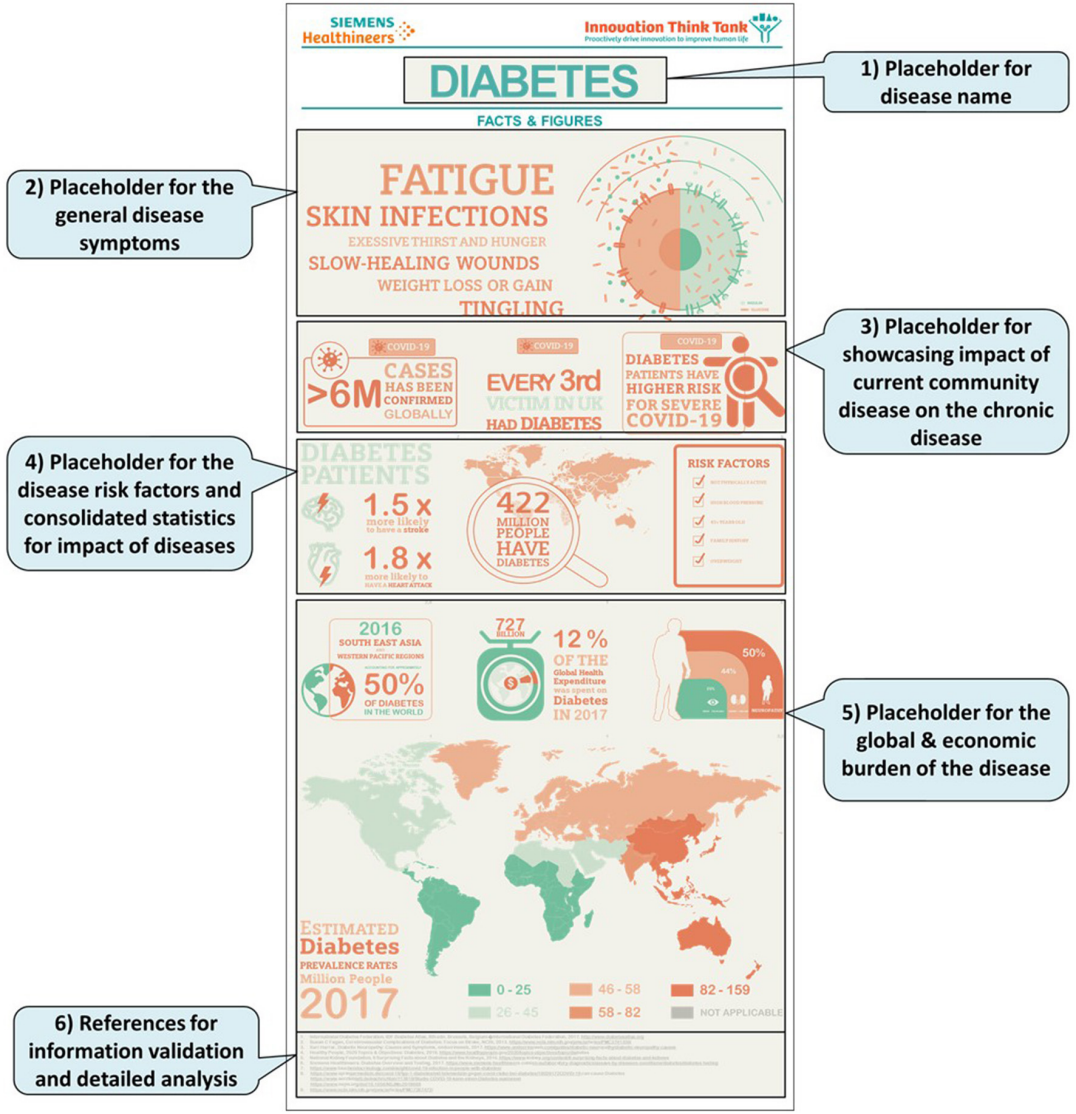
Framework template for facts and figures showcasing (1) General disease symptoms. (2) Impact of community diseases on chronic disease. (4) Risk factors and consolidated statistics of disease impact. (5) statistics of global & economic burden.

**Figure 4:**
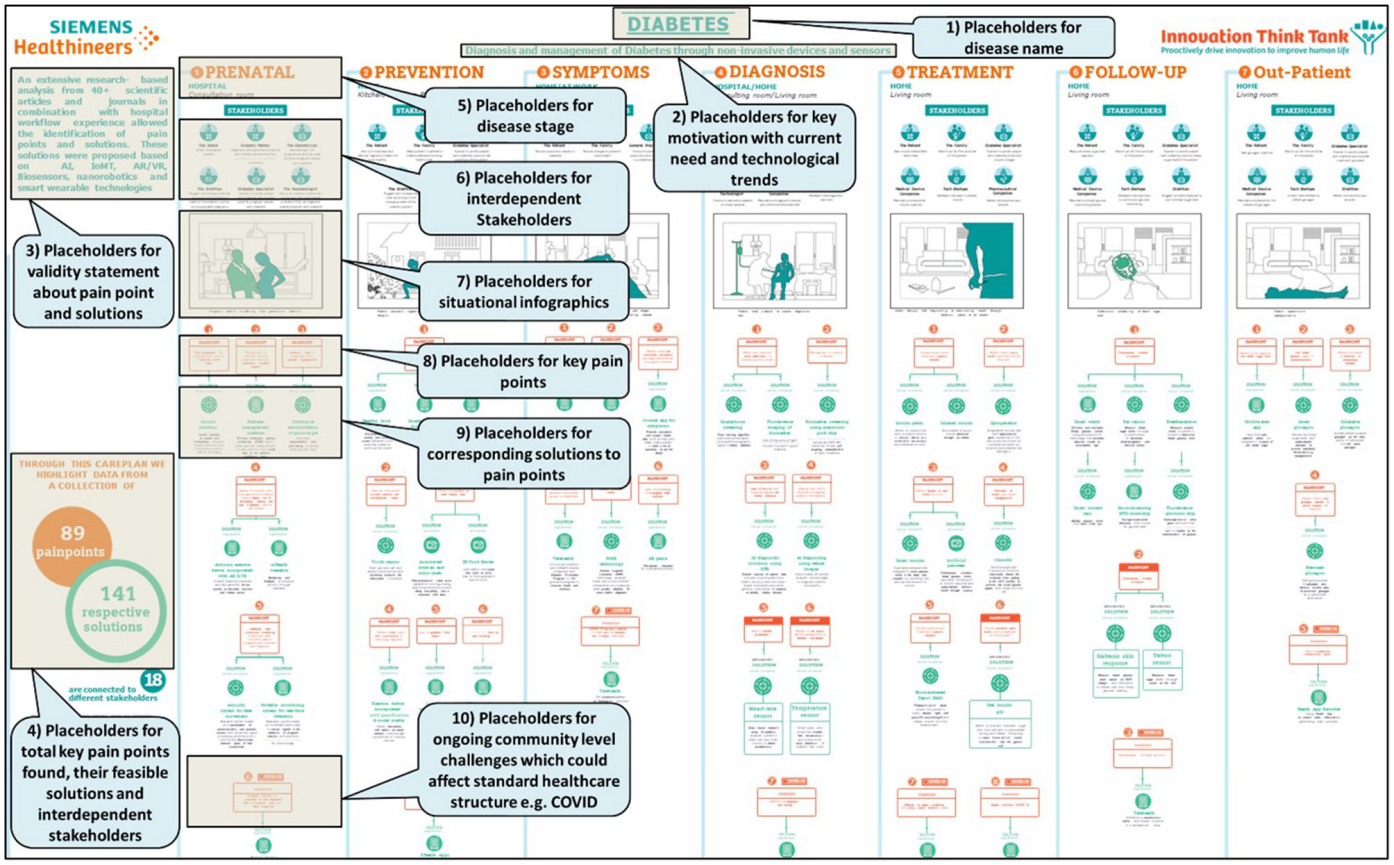
Disease pathway framework in generalized format explaining the placement of framework elements.

Thus, proposed disease pathway framework was developed comprising of the infographics which contained the facts and figures for disease, and the care plan which showed the patient journey throughout the disease progression as described above, interdependent stakeholders involved in each stage, their pain points, interdependencies between the stages, and solutions based on customer needs. For each disease, the information collected was consolidated into disease pathway framework template, including infographics and care plan stages. The care plan model described in Figures 3, 4 is the skeletal form of the disease pathway framework, where user can include the key findings. During the usability of the model for specific chronic disease key finds can be placed in their relevant placeholders. Number of pathway stages to used depends on the nature and need for the disease.

To showcase an example of a care plan taking CAD into consideration a generalized scenario was created, highlighting some pain points and potential solutions defined in each stage of the care plan. We took a use case of middle-aged man, whose mother was a smoker, has an unhealthy diet and sedentary lifestyle (33, 34). A prehospital assessment revealed a high risk for heart disease. He experiences sudden chest pain and is admitted to the hospital, where he is diagnosed with myocardial infarction, along with a positive test for COVID 19 disease. This made it more complicated to understand the exact diagnosis or cause for his condition (35, 36). Hence, it became difficult to implement an appropriate treatment plan. Moreover, lack of rehabilitation exercises and shortage of medical staff due to pandemic, delayed his recovery. To avoid such pain points, technologies like artificial intelligence, machine learning, sensors and wearables, digital twin, 5G, telemedicine, virtual reality and robotics can be utilized to continuously monitor for early signs of warning, take preventive measures, aid in faster and accurate diagnosis, determine best course of treatment plan and provide robotic-assistance during rehabilitation and follow-up (37–43).

Based on the developed framework, we created disease pathways for 22 impacting diseases. The disease pathways were analyzed from the perspective of more than 20 stakeholders for the different stages of the care plan. As a result, a total of over 1,500 pain points were identified from multiple sources, including stakeholder interactions, clinical and disease workflow analyses, research, and requirements analyses of specific modalities. In addition, more than 1,900 ideas were proposed for the identified challenges. Some of these ideas are already being worked upon in ITT's research and development laboratories. The solutions were influenced by technological advances, business models, process improvements and clinical aspects. Thus, the technological solutions were distributed across three areas, namely digital technologies, which include the Internet of Medical Things (IoMT), the 5G mobile standard, artificial intelligence (AI) and blockchain; automated solutions such as robotics and sensors; and clinical innovations, which include new diagnostic methods, digital pathology, and minimally invasive procedures.

The pathways framework is a holistic approach, which institutions can use to collect their own pain points for their relevant stakeholders and come up with their customized solutions. We would also like to put a light on the ideal size for the framework templates is A3 (297 × 420 mm) or A2 (420 × 594 mm) for the best use and to visualize the consolidated view of a particular disease.

## Discussion

Based on the literature reviewed, it is evident that the global disease burden is rising and can prove to be catastrophic for the entire healthcare infrastructure. Our healthcare system is frail, and this has been proven multiple times, recently by the COVID-19 pandemic. We need a global multidisciplinary team comprising of clinical experts, medical technologists, policy makers, researchers, strategy and innovation experts, to identify the medical needs and develop innovative solutions that will deliver quality care to patients and improve outcomes, and this is what we have done in this study. Moreover, we focused on developing a common framework that can be applicable for all existing and new diseases as it provides a basic structure that can be easily used to curate disease pathways.

The disease pathway framework demonstrates the potential for incorporating some forward-thinking solutions to improve the patient experience. Predictive models can be used to identify individuals at high risk and take timely action (23). Chemoprophylaxis, vaccination, and other technological and clinical innovations may aid in disease prevention (26). Compiling information on existing diagnostic methods provides insight into the weaknesses of the current state of the art, based on which new solutions can be developed. It is important to invest in new tools and diagnostic technologies in addition to the usual strategies (44).

The disease pathway framework enables stakeholder mapping and provides important information about stakeholders' roles and influence on the different stages of the disease life cycle. For example, some of the stakeholders in the CAD care plan would be the patient, family, paramedics, nurse, cardiologist, and healthcare facility. These pathways also catalyze the identification of pain points and allow us to target the root cause efficiently. These pain points can be from the perspective of any stakeholder involved in the pathway. Understanding these challenges and stakeholder analysis helps identify areas for improvement related to clinical applications, products, processes, workflows, and patient-centered care. Mapping the portfolio in terms of disease pathways enables an organization to identify the white spots in the market. By identifying the pain points and white spots, considering the voices of key opinion leaders (KOLs) and conducting hospital visits, information can be gathered about customers' needs and product requirements. Accordingly, decision suggestions can be made, and efficient solutions can be developed. The technological innovations can provide access to critical information for various stakeholders such as patients, physicians, hospital administrators, and government agencies. This can lead to improved patient experience and saving resources such as time and money. Thus, via the disease pathways, the framework can be used for:

1. Stakeholder Analysis
2. Pain point and root cause analysis
3. Portfolio Mapping and White spot analysis
4. Product requirement definition and Decision Proposition

The disease pathways created from this framework have been used in over 30 workshops, ITT certification programs (ITTCPs), and projects. The approach was applied to optimize use in 30 product and portfolio definition projects over 2 years at Siemens Healthineers and co-creation programs with universities and hospitals. The disease pathways helped identify the potential for improvement in various medical modalities such as CT, magnetic resonance imaging (MRI), molecular imaging (MI), X-ray equipment, surgical C-arms, angiography equipment, and laboratory diagnostics, to name a few. They also helped identify new potential disruptive areas related to technology and start-ups, opportunities to expand product portfolios, optimize clinical workflow, and enable patient-centered care. However, we also have few requirement-based limitations. If we take care of the following limitations, we can achieve best usability of the proposed framework. Disease pathways need to be adapted for different regions and locations. These pathways need to be updated regularly to reflect the latest trends and challenges. The pathways represent a very general set of best practices and pain points which need to be modified based on the healthcare systems in developed, developing, and underdeveloped countries.

Developing and implementing dedicated pathways requires a highly transdisciplinary approach with a global infrastructure consisting of trusted partners. This is often a challenge with traditional innovation approaches.

## Conclusion

The disease pathway framework can be useful for healthcare providers, medical device manufacturers, start-ups, and other stakeholders to improve clinical workflows, enable patient-centered care, define product requirements, analyze gaps, and assess business impact. It provides a unique foundation for extensive collaboration among various stakeholders through information sharing and delivering high-quality solutions based on the identified problems and customer requirements.

The futuristic ideas presented here could be implemented in the hospital infrastructure in the future to solve some of the problems currently observed, such as social constraints during a pandemic, regular appointments with the appropriate experts, and improved quality of health care. A pathway for each disease can be created and extensive global clinical validation can be done after the Covid pandemic. A digital tool (web/mobile/tablet application) of the framework can be developed to overcome covid like situations.

## Data Availability Statement

The datasets presented in this study can be found in online repositories. The names of the repository/repositories and accession number(s) can be found in the article/supplementary material.

## Author Contributions

SH designed the disease pathway framework as the base for the study, conceived the original idea, and supervised and supported the project with respect to Siemens Healthineers. SH, AG, EW, SS, MM, SM, DH, and TP carried out the experiment. SH, AG, and MM wrote the manuscript with support from EW, SS, SM, DH, and TP. All authors contributed to the article and approved the submitted version.

## Funding

This study received funding from Innovation Think Tank, Siemens Healthcare GmbH, Germany. The funder had the following involvement with the study: conceptualization, development, and manuscript writing.

## Conflict of Interest

SH, AG, EW, SS, MM, SM, DH, and TP were employed by Siemens Healthineers, Erlangen, Germany.

## Publisher's Note

All claims expressed in this article are solely those of the authors and do not necessarily represent those of their affiliated organizations, or those of the publisher, the editors and the reviewers. Any product that may be evaluated in this article, or claim that may be made by its manufacturer, is not guaranteed or endorsed by the publisher.
