# Corrigendum: A Disease Pathway Framework for Pain Point Identification and Elaboration of Product Requirements Across Patient Care Plan Using Innovation Think Tank Global Infrastructure

**DOI:** 10.3389/fpubh.2022.931376

**Published:** 2022-06-29

**Authors:** Sultan Haider, Apoorva Goenka, Mohd Mahmeen, Shamlin Sunny, Thuong Phan, Syed Ali Mehdi, Dahlia Mohamed Hassan, Elena Weber

**Affiliations:** ^1^Innovation Think Tank, Siemens Healthcare GmbH, Erlangen, Germany; ^2^Innovation Think Tank, Siemens Healthcare Pvt. Ltd., Bengaluru, India; ^3^Innovation Think Tank, Siemens Healthcare GmbH, Kemnath, Germany; ^4^Innovation Think Tank, Siemens Medical Solutions Inc., Princeton, NJ, United States; ^5^Innovation Think Tank, Siemens Healthcare LLC, Dubai, United Arab Emirates

**Keywords:** disease pathways, Innovation Think Tank, care plans, chronic disease management, disease statistics, healthcare system

In the original article, there was an error. “We have provided all developed 22 impacting disease pathways in pdf format as the **Supplementary Files** because of the size constraints for the journal publication.”

A correction has been made to **Results**, in the last paragraph:

“The pathways framework is a holistic approach, which institutions can use to collect their own pain points for their relevant stakeholders and come up with their customized solutions.”

We received some important feedback regarding the **Supplementary Files** that they should be removed as precautionary measure for avoiding misunderstanding about the data presented, which need to be regularly updated. Therefore, for important reasons and on urgent basis, we request that the **Supplementary Files** should be removed. The removal of the **Supplementary Files** does not change the subject matter of the proposed framework.

The authors apologize for this error and state that this does not change the scientific conclusions of the article in any way. The original article has been updated.

## Publisher's Note

All claims expressed in this article are solely those of the authors and do not necessarily represent those of their affiliated organizations, or those of the publisher, the editors and the reviewers. Any product that may be evaluated in this article, or claim that may be made by its manufacturer, is not guaranteed or endorsed by the publisher.

